# Anti-Inflammatory and Analgesic Effects of Curcumin Nanoparticles Associated with Diclofenac Sodium in Experimental Acute Inflammation

**DOI:** 10.3390/ijms231911737

**Published:** 2022-10-03

**Authors:** Ioana Boarescu, Raluca Maria Pop, Paul-Mihai Boarescu, Ioana Corina Bocșan, Dan Gheban, Ruxandra-Mioara Râjnoveanu, Armand Râjnoveanu, Adriana Elena Bulboacă, Anca Dana Buzoianu, Sorana D. Bolboacă

**Affiliations:** 1 Department of Medical Informatics and Biostatistics, Iuliu Haţieganu University of Medicine and Pharmacy Cluj-Napoca, Louis Pasteur Street, No. 6, 400349 Cluj-Napoca, Romania; 2 Department of Pharmacology, Toxicology and Clinical Pharmacology, Iuliu Haţieganu University of Medicine and Pharmacy Cluj-Napoca, Gheorghe Marinescu Street, No. 23, 400337 Cluj-Napoca, Romania; 3 Department of Pathological Anatomy, Iuliu Haţieganu University of Medicine and Pharmacy Cluj-Napoca, Clinicilor Street, No. 3-5, 400006 Cluj-Napoca, Romania; 4 Department of Palliative Medicine, Iuliu Haţieganu University of Medicine and Pharmacy Cluj-Napoca, B.P. Hașdeu Street, No. 6, 400371 Cluj-Napoca, Romania; 5 Department of Occupational Medicine, Iuliu Haţieganu University of Medicine and Pharmacy Cluj-Napoca, Clinicilor Street, No. 3-5, 400006 Cluj-Napoca, Romania; 6 Department of Pathophysiology, Iuliu Haţieganu University of Medicine and Pharmacy Cluj-Napoca, Victor Babeş Street, No. 2-4, 400012 Cluj-Napoca, Romania

**Keywords:** curcumin, nanoparticles, diclofenac sodium, carrageenan, inflammation, cytokines

## Abstract

The present study evaluated the anti-inflammatory and analgesic effects of conventional curcumin (cC) and curcumin nanoparticles (nC) associated with diclofenac sodium (D) in experimental acute inflammation (AI) induced by carrageenan administration. Seven groups of eight randomly selected Wistar-Bratislava white rats were evaluated. One group was the control (C), and AI was induced in the other six groups. The AI group was treated with saline solution, the AID group was treated with D, the AIcC200 and AInC200 groups were treated with cC and nC, respectively, while AIcC200D and AInC200D were treated with cC and nC, respectively, both associated with D. Conventional curcumin, nC, and D were administered in a single dose of 200 mg/kg b.w. for cC and nC and 5 mg/kg b.w. for D. Association of cC or nC to D resulted in significant antinociceptive activity, and improved mechanical pressure stimulation and heat thresholds at 3, 5, 7 and 24 h (*p* < 0.03). The association of cC and nC with D (AIcC200D and AInC200D groups) showed significantly lower plasma and tissue levels of tumor necrosis factor-α (TNF-α), interleukin-6 (IL-6), and interleukin-1β (IL-1β) up to 2.5 times, with the best results in the group who received nC. Moreover, AInC200D presented the least severe histopathological changes with a reduced level of inflammation in the dermis and hypodermis. The combination of nC to D showed efficiency in reducing pain, inflammatory cytokines, and histological changes in acute inflammation.

## 1. Introduction

Inflammation is a broad and ancient medical term referring to a set of classic signs and symptoms, including pain, edema, hyperemia, warmth, and loss of function [1]. It is characterized by a group of complex changing responses to tissue injury primarily caused by harmful stimuli, such as pathogens, physical agents, chemical compounds, or damaged cells [2].

Carrageenan is a pro-inflammatory polysaccharide used to induce local inflammation (paw edema) [2,3]. The release of bradykinin, serotonin, histamine, and prostaglandins under cyclooxygenase enzymes (COX) occurs in the early phase of inflammation. In the late phase, prostaglandin generation is continued together with neutrophil infiltration and release of pro-inflammatory cytokines such as tumor necrosis factor (TNF-α) and interleukin-1 β (IL-1β) [4]. The overproduction of neutrophil-derived free radicals and nitric oxide (NO) is also involved in the delayed phase of carrageenan-induced acute inflammation [5]. It was suggested that drugs targeting the COX enzyme, pro-inflammatory expression (e.g., inducible nitric oxide synthase; iNOS), and free radical formation might better control the inflammation process [6].

Non-Steroidal Anti-Inflammatory Drugs (NSAIDs) are one of the most commonly prescribed medications for acute and chronic inflammation. Their major therapeutic actions are related to their ability to block certain prostaglandins (PGs) synthesis through cyclooxygenase enzymes (COX-1 and COX-2) inhibition [7]. Cyclooxygenase enzyme 1 (COX-1) is expressed in normal cells and produces PGs and thromboxane A2, which control renal homeostasis, platelet aggregation, and mucosal barrier in the gastrointestinal tract and possess other physiological functions. Cyclooxygenase enzyme 2 (COX-2) is induced in inflammatory cells and produces PGs related to inflammation, pain, and fever. The inhibition of COX-2 most likely represents the desired effect of NSAIDs by providing anti-inflammatory, analgesic, and antipyretic responses; inhibition of the COX-1 enzyme plays a major role in the undesired side effects such as injury to the gastrointestinal mucosa [8]. Therefore, in some circumstances, NSAID administration may cause acute renal failure, gastrointestinal ulcers, hypertension, or serious cardiovascular events (such as stroke or acute myocardial infarction). These adverse effects may be prevented first by limiting NSAID dosage and duration of administration and also by performing risk assessments for each patient depending on the associated pathology [9].

Diclofenac (D) is a nonselective NSAID widely used as an anti-inflammatory, antipyretic, and analgesic drug but it has a reduced bioavailability due to hepatic first-pass metabolism and associates many adverse effects on gastrointestinal, hepatic, renal, and cardiovascular systems [10,11].

Curcumin is a major bioactive phytochemical in the rhizome of turmeric (*Curcuma longa* L.). It has advantageous pharmacological properties, including antioxidant, anti-inflammatory, anticancer, and anti-infective properties but its utility in clinical practice is limited due to its low aqueous solubility, poor absorption, and metabolic instability, which lead to poor oral bioavailability [12].

We previously demonstrated that conventional curcumin (cC) and curcumin nanoparticles (nC) potentiate the antiedematous effects and antioxidant activity of diclofenac in carrageenan-induced paw edema, with better results for nC [13]. The present study aimed to evaluate the anti-inflammatory and analgesic effects of conventional curcumin and curcumin nanoparticles associated with diclofenac sodium in experimental acute inflammation induced by carrageenan administration.

## 2. Results

### 2.1. Motility Test

**Figure 1:**
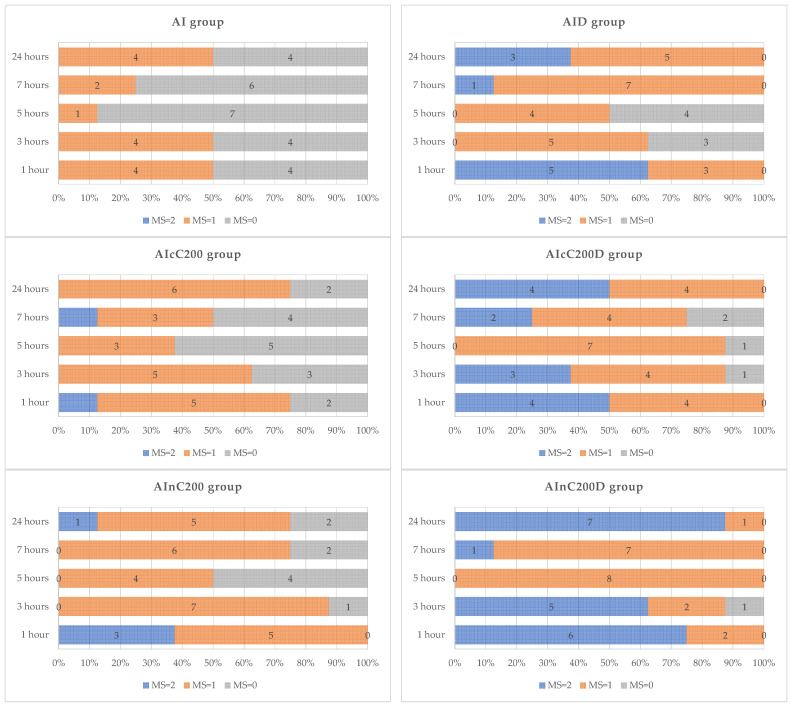
Effects of diclofenac sodium (D), conventional curcumin (cC), and curcumin nanoparticles (nC) on motility score (MS). Interpretation: MS = 2 indicates no motility problems; MS = 1 indicates some motility problems (walk with difficulty but with the toe of the inflamed paw touching the floor), and MS = 0 indicates motility problems (walk with difficulties and avoided touching the right hind paw to the floor). C—control (*n* = 8); AI—Acute inflammation (*n* = 8); D—Diclofenac sodium (*n* = 8); cC—conventional curcumin solution (200 mg/kg b.w.; *n* = 8); nC—curcumin nanoparticles solution (200 mg/kg b.w.; *n* = 8).

Rats from the C group exhibited, without any exception, scores of 2 to all determinations (Figure 1, Table S1). Rats from the AI group presented the lowest motility score starting from the first hour after carrageenan administration up to 24 h (Figure 1). Conventional curcumin and curcumin nanoparticles improved the motility score, with a better motility score for nC, which was similar to D (Figure 1, Table S1). Better walking abilities of the rats in the AIcC200D and AInC200D groups were observed than in the D group, with the highest motility score in the AInC200D group (Figure 1).

### 2.2. Paw Pressure Test

**Figure 2:**
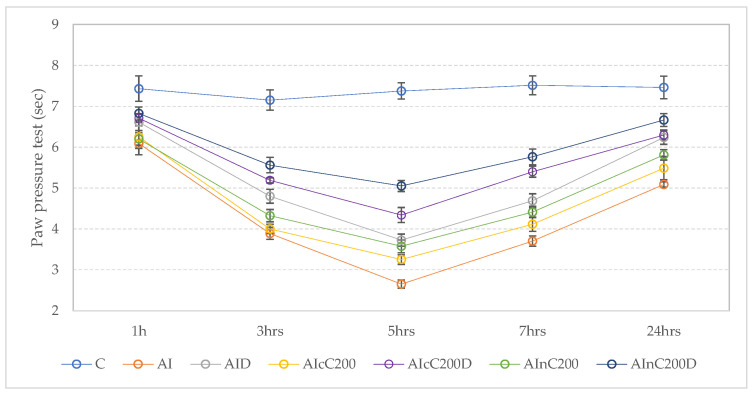
Effects of diclofenac sodium (D), conventional curcumin (cC) and curcumin nanoparticles (nC) on nociceptive thresholds. Circles represent the mean value of each group; whiskers represent the value of the SEM (standard error of the mean). C—control (*n* = 8); AI—Acute inflammation (*n* = 8); D—Diclofenac sodium (*n* = 8) ; cC—conventional curcumin solution (200 mg/kg b.w.; *n* = 8); nC—curcumin nanoparticles solution (200 mg/kg b.w.; *n* = 8).

The AI group’s rat presented a statistically reduced threshold in the paw pressure test compared to the control group at 3, 5, 7, and 24 h (*p* < 0.0001, Figure 2). The nociceptive thresholds presented a progressive decrease, reaching the minimum values 5 h after AI induction. Diclofenac administration improved nociceptive thresholds but no statistical significance was found compared to the AI group, at any follow-up measurements (*p* > 0.0753, Figure 2). Rats treated with cC and nC had a better but not statistically significant (*p* > 0.8711, Figure 2) analgesic response compared to the AI group, which was less efficient than diclofenac alone (Figure 2). Moreover, rat responses from the above-mentioned groups were significantly reduced compared to the C group (*p* ≤ 0.0190, Figure 2), starting with the first hour after AI induction. When cC and nC were associated with D, rats had a significantly better nociceptive response at 3, 5, 7, and 24 h, with thresholds significantly higher than in the AI group (*p* ≤ 0.0285, Figure 2). However, they were not significantly different than the C group (*p* ≥ 0.6693, Figure 2), with the best results observed when D was associated with nC (AInC200D group, Figure 2).

The mean and standard deviations for each group on paw pressure test are presented in Table S2, while the *p*-values for comparisons between groups are presented in Table S3.

### 2.3. Hot Plate Test

**Figure 3:**
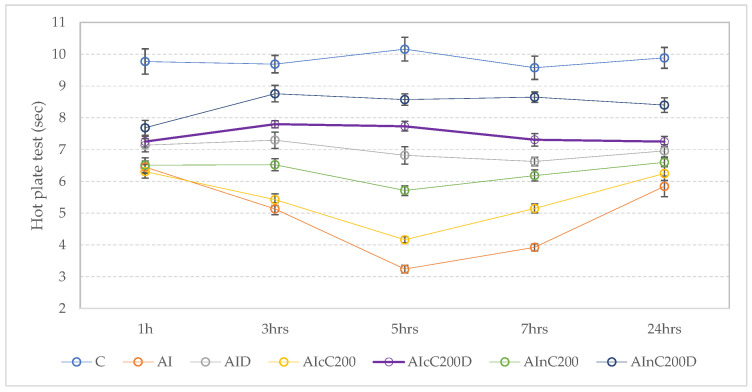
Effects of diclofenac sodium (D), conventional curcumin (cC), and curcumin nanoparticles (nC) on reactions time to the heat. Circles represent the mean value of each group; whiskers represent the value of the SEM (standard error of the mean). C—control (*n* = 8); AI—Acute inflammation (*n* = 8); D—Diclofenac sodium (*n* = 8) ; cC—conventional curcumin solution (200 mg/kg b.w.; *n* = 8); nC—curcumin nanoparticles solution (200 mg/kg b.w.; *n* = 8).

Carrageenan administration led to a reduction in reaction time to the heat, starting from the first hour. In the AI group, this reduction was progressive and statically significant compared to the C group (*p* ≤ 0.0002, Figure 3), with the minimum values at 5 h (Figure 3).

Diclofenac improved the time response in all measurements but the improvement was not statistically significant compared to the AI group (*p* ≥ 0.0545, Figure 3). Conventional curcumin and curcumin nanoparticles showed an elevation in pain threshold compared to the AI group but without statistical significance (*p* ≥ 0.3832, Figure 3). Compared to the C group, both cC and nC groups had significantly reduced thresholds (*p* ≤ 0.0052, Figure 3). Association of cC or nC to D resulted in significant antinociceptive activity and improved thresholds to the heat at 3, 5, and 7 h for both groups, and at 24 h for AInC200D, when compared to the AI group (*p* < 0.0175, Figure 3). A similar reaction time to the heat was observed in the AInC200D group and C group (*p* > 0.9999, Figure 3).

The mean and standard deviation for each group on the hot plate test responses are presented in Table S4. The results of comparisons between groups are expressed as *p*-values shown in Table S5.

### 2.4. Inflammatory Cytokines

**Figure 4:**
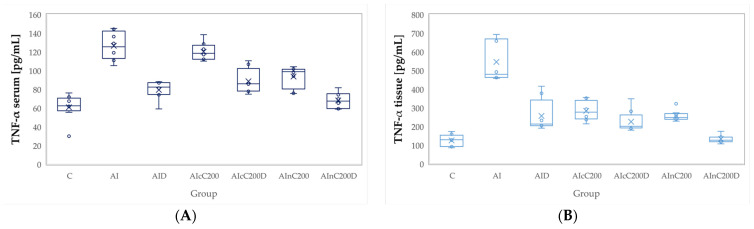
Variation by groups of TNF-α (Tumor necrosis factor α): (**A**) in the plasma (**B**) in the tissue. C—control; AI—Acute inflammation; D—Diclofenac; cC—conventional curcumin solution (200 mg/kg b.w.); nC—curcumin nanoparticles solution (200 mg/kg b.w).

**Figure 5:**
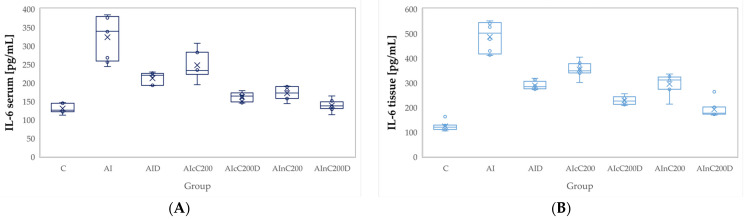
Variation by groups of IL-6 (Interleukin 6): (**A**) in the plasma (**B**) in the tissue. C—control; AI—Acute inflammation; D—Diclofenac; cC—conventional curcumin solution (200 mg/kg b.w.); nC—curcumin nanoparticles solution (200 mg/kg b.w.).

**Figure 6:**
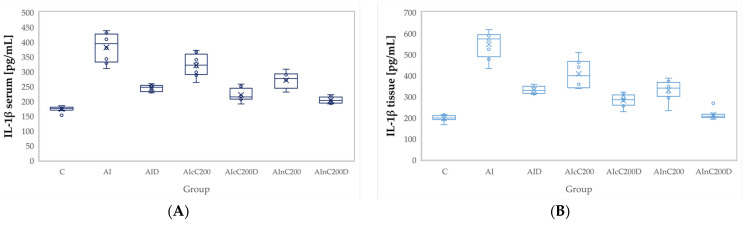
Variation by groups of IL-1β (Interleukin 1β): (**A**) in the plasma (**B**) in the tissue. C—control; AI—Acute inflammation; D—Diclofenac; cC—conventional curcumin solution (200 mg/kg b.w.); nC—curcumin nanoparticles solution (200 mg/kg b.w.).

Administration of carrageenan led to increased plasma and tissue levels of all evaluated pro-inflammatory cytokines, namely tumor necrosis factor (TNF-α), interleukin 6 (IL-6), and interleukin 1β (IL-1β). All these cytokines were significantly increased in the AI group compared to the C group (*p* < 0.0001, Figure 4, Figure 5 and Figure 6). Diclofenac sodium administration significantly reduced the plasma levels of TNF-α (*p* = 0.0170, Figure 4). Tissue levels of TNF-α and both plasma and tissue levels of IL-6 and IL-1β were also reduced by D administration compared to the AI group but statistical significances were not reached (*p* ≥ 0.14, Figure 4, Figure 5 and Figure 6).

The cC alone group offered a slightly reduced anti-inflammatory effect, proved by the reduction of the plasma and tissue levels of all three cytokines, insignificantly compared to the AI group (*p* > 0.9999, Figure 4, Figure 5 and Figure 6). Rats from the AIcC200 group had statistically higher levels of all evaluated cytokines compared to the control group (*p* ≤ 0.0044, Figure 4, Figure 5 and Figure 6). Similar results but with lower levels than the AIcC200 group of the evaluated pro-inflammatory cytokines were observed when nC was administered alone (Figure 4, Figure 5 and Figure 6).

Tissue levels of TNF-α and both plasma and tissue levels of IL-6 and IL-1β were reduced when cC was added to D and then D alone (Figure 4B, Figure 5, and Figure 6) without reaching statistical significance (*p* > 0.05). When the AIcC200D group was compared to the AI group, statistical significance was reached for the above-mentioned parameters (*p* ≤ 0.0212). The combination of nC and D improved plasma and tissue levels of all three cytokines compared to the AI group (*p* ≤ 0.0002, Figure 4, Figure 5 and Figure 6). Moreover, the cytokine levels in the AInC200D group were similar to those of the control group (*p* ≥ 0.9999, Figure 4, Figure 5 and Figure 6).

The summary statistics of pro-inflammatory cytokines by each group are presented in Table S6, with the results of the comparison between groups presented in Table S7.

**Figure 7:**
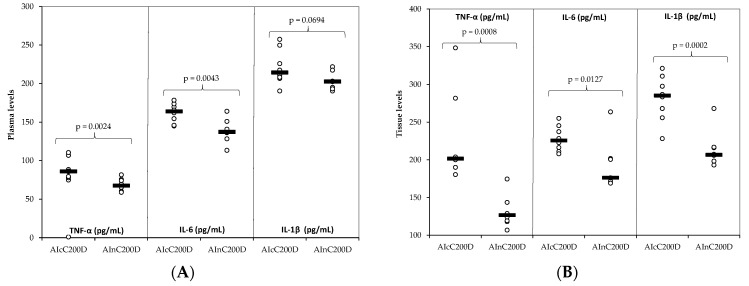
The increase in the therapeutic effect of diclofenac D when combined with conventional curcumin (cC) and curcumin nanoparticles (nC): (**A**) in the plasma (**B**) in the tissue. C—control; AI—Acute inflammation; D—Diclofenac; cC—conventional curcumin solution (200 mg/kg b.w.); nC—curcumin nanoparticles solution (200 mg/kg b.w.). The dots are the primary data and the line indicates the median value.

Excepting the plasma levels of IL-1β where the difference had only a tendency to statistical significance (*p* < 0.10), nC enhances the therapeutic effect of diclofenac on all evaluated markers, both in plasma and tissue significantly better than cC (Figure 7).

### 2.5. Quantification of Plasma Curcumin

**Figure 8:**
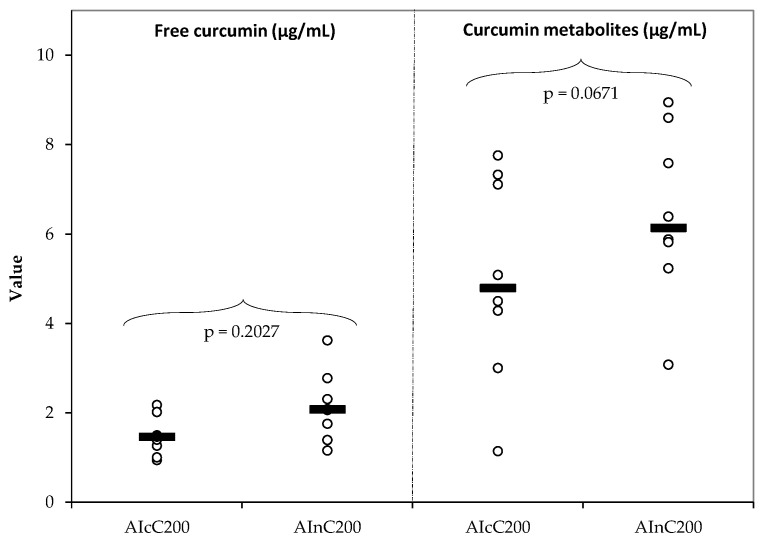
Variation of cC and nC: free curcumin before hydrolyzation and curcumin’s metabolites after hydrolyzation. AI—Acute inflammation; cC—conventional curcumin solution (200 mg/kg b.w.); nC—curcumin nanoparticles solution (200 mg/kg b.w.). The dots are the primary data and the line indicates the median value.

Curcumin levels before hydrolyzation were 1.4-fold higher in rats treated with nC than cC, while plasma levels of its metabolites were 1.3-fold higher after nC administration compared to cC, with only a tendency to statistical significance for curcumin metabolites (Figure 8).

### 2.6. Histopathological Examination

**Figure 9:**
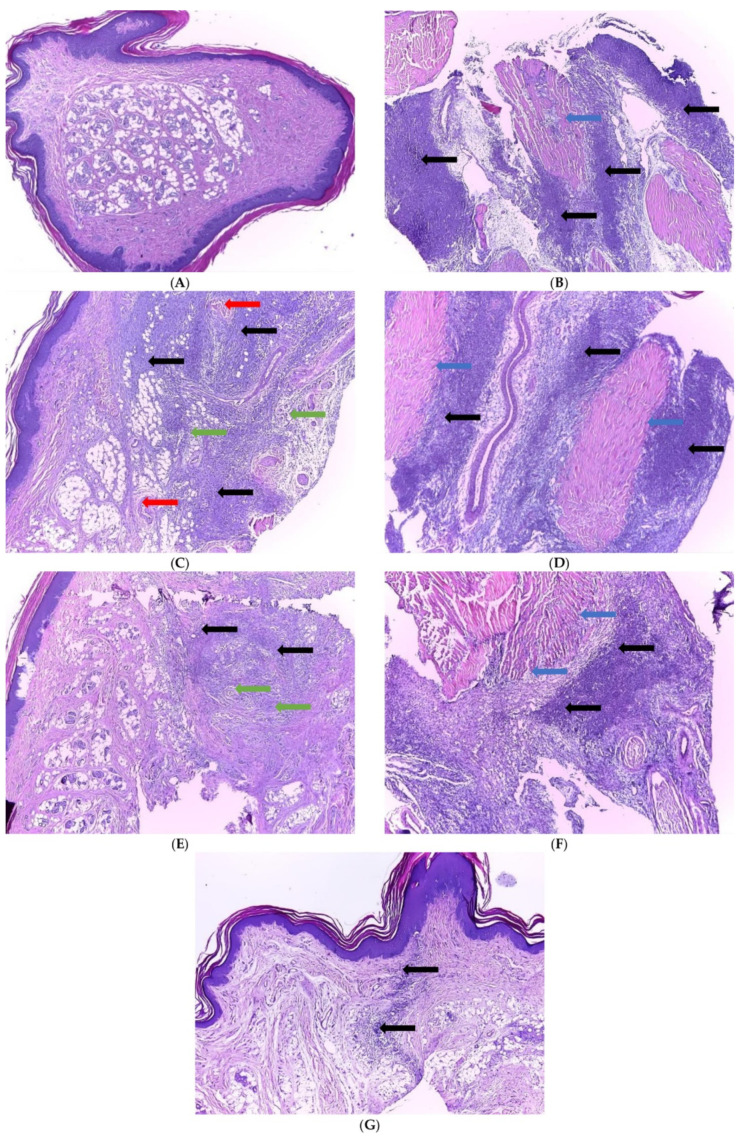
Histopathological examinations of the fragments from right-hind paw: (**A**) C-normal architecture of dermis and hypodermis of the plantar surface of the foot; (**B**) AI-acute purulent inflammation in the dermis, hypodermis (**black arrow**), with an extension on the underlying muscular tissue (**blue arrow**); (**C**) AID-dermis and hypodermis with signs of acute purulent inflammation (**black arrow**), micro-abscess formation (**green arrow**), and micro-thrombi (**red arrow**) in several vascular lumens; (**D**) AIcC200-acute purulent inflammation in the dermis, hypodermis (**black arrow**), with the extension on the underlying muscular tissue (**blue arrow**); (**E**) AIcC200D-inflammation focalized in the hypodermis (**black arrow**) associated with microabscess formation (**green arrow**); (**F**) AInC200-acute purulent inflammation in the dermis, hypodermis (**black arrow**), with an extension on the underlying muscular tissue (**blue arrow**); (**G**) AInC200D-reduced level of inflammation on dermis and hypodermis (**black arrow**).

Histopathological examination of the hind paw of the control group showed normal structure and architecture without signs of inflammation in the dermis and hypodermis (Figure 9A). In the AI group, severe inflammation was observed after carrageenan administration, characterized by acute purulent inflammation in the dermis and hypodermis, extending to the underlying muscular tissue (Figure 9B). In the D group, 24 h after the AI induction, the histological examination revealed signs of acute purulent inflammation in the dermis and hypodermis with microabscess formation and micro-thrombi in several vascular lumens (Figure 9C). Conventional curcumin and nC administrated alone did not influence the histopathological aspects of the hind paw, highlighted by the same histopathological changes observed in the AI group: acute purulent inflammation in the dermis and hypodermis, with extension to the underlying muscular tissue (AIcC200 group in Figure 9D and AInC200 in Figure 9F). Conventional curcumin associated with diclofenac (AIcC200D group) led to inflammation focalized only in the hypodermis but associated with microabscess formation (Figure 9E). Less severe histopathological changes were observed in groups treated with nC and D (AInC200D group) as rats presented reduced inflammation of the dermis and hypodermis (Figure 9G).

## 3. Discussion

The results of our study demonstrated that the association of conventional curcumin and curcumin nanoparticles to diclofenac sodium could potentiate the analgesic and anti-inflammatory effects of diclofenac sodium in reduced doses, with the best results obtained for curcumin nanoparticles.

Curcumin (both cC and nC) potentiates the anti-edematogenic activity of diclofenac [13], leading to the improvement of the motility score regardless of its form as cC or nC (Figure 1).

The paw pressure test helps to assess nociceptive thresholds to mechanical pressure stimulation, as a measure of mechanical hyperalgesia [14]. Diclofenac has already proved to have an analgesic effect and to increase the withdrawal threshold in the paw pressure test [15]. Higher doses of diclofenac sodium (e.g., 20 mg/kg b.w.) provided higher tolerance of mechanical hyperalgesia compared to the control group [16,17]. In our study, the dose of diclofenac sodium (5 mg/kg b.w.) was observed to have a limited effect on response thresholds to mechanical pressure stimulation (Figure 2), most probably explained by the reduced dose of the active substance. Curcumin administration (cC and nC) also had an analgesic effect (Figure 2). Its analgesic effect could be explained by the stimulation of protein expression of β-endorphin and encephalin, which are endogenous opioid peptides [18]. We observed that the association of cC and nC to D has better analgesic effects (Figure 2), most probably because the combination acts on two mechanisms to reduce the pain threshold.

The hot plate test assesses the central activity since the response reflex is mediated by supraspinal centers. Even more, it was stated that any agent that causes a prolongation of the hot plate latency using this test must act centrally [19]. Diclofenac, like other NSAIDs, has an analgesic effect characterized by elevation of time to paw withdrawal to thermal stimuli (Figure 3), which is attributed to inhibition of the COX-mediated biosynthesis of PGs, with peripheral and central sites of action [20]. As regards curcumin administration, it was previously reported as having a good analgesic effect on thermal injury pain, an effect that could be mediated through suppression of inflammatory protein-induced pain perception (peripheral action) [21], but since it prolonged the response to the hot plate test, it may also have a central activity.

Tumor necrosis factor α (TNF-α) is an inflammatory cytokine produced by macrophages/monocytes during acute inflammation [22]. It participates in leukocyte adhesion to the epithelium by expressing adhesion molecules, vasodilatation, and edema formation [23]. In carrageenan-induced mouse paw edema, TNF-α promoted collateral cytotoxicity by acting as a stimulator of prostaglandin synthesis, an activator of the NF-κB signal transduction pathway, as NO formation inducer, and a stimulator of neutrophil migration [24].

Interleukin-6 (IL-6) is a pro-inflammatory cytokine produced by many types of cells, expressed during states of cellular stress, such as cancer, infection, wound sites, and inflammation [25]. In the inflammation process, IL-6 is produced at the site of inflammation in the initial stage and moves to the liver through the bloodstream. It leads to the rapid synthesis of an extensive range of acute phase proteins such as C-reactive protein (CRP), fibrinogen, haptoglobin, serum amyloid A, and α1-antichymotrypsin [26]. In carrageenan-induced paw edema, increased levels of IL-6 at the site of inflammation facilitate leukocyte recruitment and mediate edema formation and inflammatory pain hypersensitivity [27].

Interleukin-1 α and β are prototypic pro-inflammatory cytokines that play key roles in acute and chronic inflammatory, autoimmune disorders, or other pathologies, as they have pleiotropic effects on various cells [28,29]. Interleukin-1β is a potent mechanical and thermal hyperalgesic agent when injected into peripheral tissues [30]. Intraplantar injection of an inflammatory agent, such as carrageenan, produces mechanical or thermal hyperalgesia associated with an upregulation of IL-1β and other inflammatory cytokines in the inflamed tissue and the dorsal root ganglia [31]. It was also suggested that IL-1β inhibition could represent a broad-acting and efficacious method for managing pain and inflammation across various conditions such as gout, rheumatoid arthritis, or neuropathic pain [28].

Diclofenac, as an NSAID drug, may reduce the levels of pro-inflammatory cytokines (Figure 4, Figure 5 and Figure 6) through COX enzyme inhibition. Consequently, it may also reduce the production of prostaglandins and thromboxanes, which participate in the inflammatory response [32]. Moreover, it was observed that NSAIDs might interact with transcriptional factors and affect the production of cytokines by inhibiting the levels of TNF-α and IL-6 [33]. The reduced dose of diclofenac sodium might explain the limited reduction of all three studied pro-inflammatory cytokines (Figure 4, Figure 5 and Figure 6).

Curcumin administration was observed to prevent the elevation of interleukin IL-1β, IL-6, and TNF-α (Figure 4, Figure 5 and Figure 6) since curcumin can reduce inflammatory responses by interfering with NF-κB activation, a critical pathway in the regulation of transcription of pro-inflammatory related genes [34]. The increased anti-inflammatory effect of nC can be explained by its increased tissue distribution [35,36,37] compared to cC.

The beneficial effects of curcumin are limited due to its hydrophobic characteristics, as curcumin is not soluble in water, is poorly absorbed in the small intestine, and it has an extensive reductive and conjugative metabolism in the liver [38]. The use of biocompatible water-based polymers offers enhanced solubility, stability, pharmacological activity, and bioavailability, and avoids physical and chemical degradation of curcumin [39].

Following oral administration, curcumin is rapidly converted into glucuronide and sulfate metabolites. On intravenous or intraperitoneal injection, curcumin is also reduced to dihydrocurcumin (DHC), tetrahydrocurcumin (THC), and hexahydrocurcumin (HHC), of which THC is the predominant species. Other metabolites of curcumin are curcumin glucuronide-sulfate and glucuronide conjugates of THC and HHC [40]. Higher levels of plasma-free curcumin and its metabolites (curcumin glucuronide and curcumin sulfate) quantified after nC administration (Figure 8) are the results of higher bioavailability. This is attributed to the increased absorption of curcumin nanoparticles through the gastrointestinal tract offered by the encapsulation of the active compound in nano-carriers [41]. As a result, the therapeutical effect of D is significantly increased when nC is added as compared to the addition of cC (Figure 7).

In the present study, no significant differences were found between the AI group and the groups pretreated with cC or nC regarding the histopathological changes, as all the rats presented purulent inflammation in the dermis and hypodermis, with an extension on the underlying muscular tissue (Figure 9B,D,F). Diclofenac sodium slightly reduced the inflammatory process. Limited effects of diclofenac sodium on the histopathological changes in the carrageenan-induced rat paw edema model were also reported by Abdelhameed et al. [42]. Even if they used a higher dose of diclofenac sodium (30 mg/kg b.w.), Abdelhameed et al. reported that the oral or topical administration of diclofenac did not have a curative effect [42]. The presence of the micro-thrombi in some vascular lumens observed in the AID group might be due to the prothrombotic effects of diclofenac [43]. The association of cC with D reduced the inflammation level as it was focalized in the hypodermis associated with microabscess formation (Figure 9E), most probably because curcumin can enhance the anti-inflammatory effect of diclofenac sodium through inhibition of prostaglandin synthesis via the cyclooxygenase pathway [44]. Curcumin nanoparticles were already demonstrated to provide superior anti-inflammatory effects in an animal model of acute and chronic inflammations when given orally [45]. It is suggested that by reducing curcumin’s particle size to the nanoscale, the bioavailability is improved by enhancing the solubility [45]. Therefore, much of the active compound reaches the tissue and potentiates the diclofenac’s anti-inflammatory effects. It could explain the best histological results obtained when nC was associated with D, with reduced inflammation of the dermis and hypodermis (Figure 9G).

In an experimental study performed by Nurullahoglu et al. [46], curcumin suspension in a dose of 400 mg/kg b.w. prepared in 5% ethanol solution, proved more potent than diclofenac alone in the early phase of pain on formalin test. Jain et al. [47] observed that curcumin–diclofenac conjugate enhanced the bioavailability of curcumin more than five-fold and alleviated the symptoms of arthritis as compared to both diclofenac and curcumin in a streptococcal cell wall-induced arthritis model. Moreover, one study performed by De Paz-Campos revealed that the diclofenac–curcumin combination might have therapeutic advantages for the clinical treatment of inflammatory pain [48]. Another experimental study concluded that curcumin nanoparticles present better anti-inflammatory effects of carrageenan-induced inflammation than conventional curcumin [45].

To our knowledge, no evaluation of the additional anti-inflammatory and analgesic effects of cC or nC to diclofenac sodium in experimental acute inflammation induced by carrageenan had been previously published. Moreover, in this study, we also reported an HPLC-UV method for quantifying cC and nC before and after hydrolyzation of its major metabolites, curcumin glucuronide and curcumin sulfate, in rat plasma.

## 4. Materials and Methods

### 4.1. Chemicals and Drugs

Conventional curcumin (cC) and carrageenan (a high-molecular-weight sulfated pro-inflammatory polysaccharide) were purchased from Sigma-Aldrich (St. Louis, MO, USA), and curcumin nanoparticles (nC) were bought from CVI Pharma (Hanoi, Vietnam), while diclofenac sodium (D) and saline solution were purchased from a local pharmacy. For the curcumin nanoparticles, the active compound is encapsulated in biocompatible water-based polymers with a size between 30 and 100 nm. Standard curcumin, β-glucuronidase from Helix Pomatia, sulfatase from Helix pomatia, HPLC grade acetonitrile and methanol, ammonium acetate, dibasic potassium phosphate (K_2_HPO_4_), dimethylsulfoxide (DMSO) and Phosphate buffered saline (PBS) tablets were purchased from Merk (Darmstadt, Germany).

### 4.2. Animals and Experimental Design

**Figure 10:**
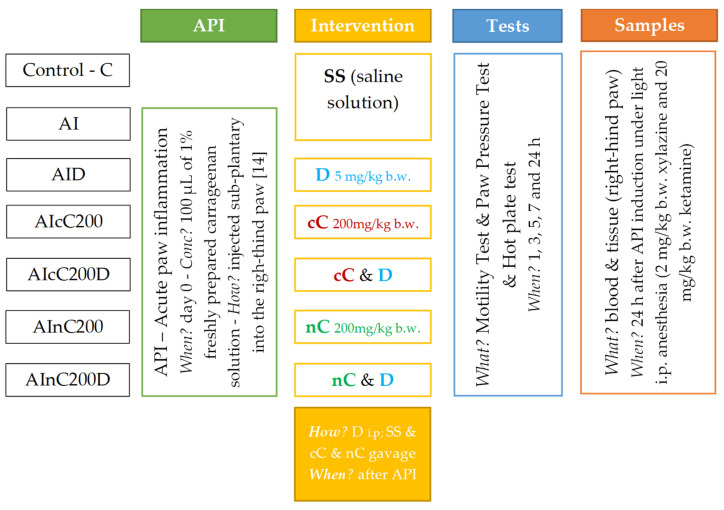
Flowchart demonstrating the study groups and interventions.

Fifty-six (56) ten-week-old white male Wistar-Bratislava rats (300 ± 10 g) were randomly divided into eight groups of seven animals each, following the flowchart from Figure 10, explained in detail in our previous research [13].

### 4.3. Time Point Measurements

The motility test, the paw pressure test and the hot plate test were performed at 1, 3, 5, 7, and 24 h after carrageenan injection.

#### 4.3.1. Motility Test

The motility pattern of the rats from all groups was noted for 5 min. Each rat was scored with 2 if the rat walked easily; 1 if the rat walked with little difficulty but with the toe of the inflamed paw touching the floor; and 0 if the rat had difficulties in walking and avoided touching the right hind paw to the floor [49].

#### 4.3.2. Paw Pressure Test

An analgesia meter (Ugo Basile, Milan, Italy) was used for the mechanical nociceptive response measurement. For this test, plantar mechanical pressure was applied linearly by increasing the mechanical force to the rat’s right-hind paw. The value of 500 g was set as cut-off pressure and the retraction of the paw of the rat’s squeak was recorded as the latency response [50].

#### 4.3.3. Hot Plate Test

The hot plate test is a therm analgesic method that measures the threshold response to pain. This thermal test was selected because it is sensitive to strong analgesics and limits tissue damage because of a cut-off point that is usually applied to reduce the time the rats spend on the hot plate [19]. The heat sensitivity of the paw was assessed using an Ugo Basile hot plate (Milan, Italy) heated to 55 °C. During the whole test, the plate was kept at a constant temperature of 55 ± 0.1 °C. Rats from all groups were placed on the hot plate, and their reactions were observed. Time of latency was defined as the time between the zero moment when the rat was placed on the hot plate surface and the moment when the rat licked its hind paw or jumped off to avoid thermal pain. Thirty seconds was the cut-off time chosen to minimize tissue damage [51].

### 4.4. Blood Samples

Under light anesthesia with ketamine (20 mg/kg b.w., i.p.) and xylazine (2 mg/kg b.w., i.p.), the blood samples were collected in heparinized tubes (Startstedt AG and Co., Nümbrecht, Germany) from the retro-orbital plexuses of each rat, at 24 h after AI induction. Plasma samples were obtained by centrifugation at 4 °C for 20 min at 1620 (×g). The obtained plasma was further transferred in Eppendorf tubes and kept at −80 °C until further analysis. All rats were sacrificed by an overdose of anesthetics at the end of the experiment.

### 4.5. Tissue Homogenate

Tissue samples were taken from the right-hind paw of rats from all groups immediately after scarification. The tissue samples were weighed and homogenized using an automated Witeg Homogenizer (HG-15D, Wertheim, Germany) in four volumes of phosphate-buffered saline solution, centrifuged at 1500 (×g) for 15 min at 4 °C and the resulting clear supernatant was stored used for further biochemical analysis.

### 4.6. Biochemical Assays

The plasma and tissue levels of the inflammatory cytokines (TNF-α, IL-6, and IL-1β) were measured using the enzyme-linked immunosorbent assay (ELISA) technique (Stat Fax 303 Plus Microstrip Reader, Minneapolis, MN, USA), with commercially available kits (rat TNF-α, IL-6, and IL-1β ABTS ELISA Development kits, PeproTech EC, Ltd., London, UK).

### 4.7. Quantification of Plasma Curcumin

#### 4.7.1. Preparation of Stock Solutions and Calibration Standards

Standard curcumin 5 mg was accurately weighed and transferred to a 5 mL amber-colored volumetric flask. The volume was made with DMSO obtaining a concentration of 1 mg/mL. This solution was diluted with DMSO to obtain a final concentration range between 0.05 and 5 μg/mL. The dilutions were also prepared in amber-colored HPLC vials and stored at −20 °C not longer than one month after preparation. Standard solutions were freshly prepared each day using the stock solution for intraday and interday analysis. Before injecting into the HPLC system, the solutions were filtered using 0.45 µm nylon filters.

#### 4.7.2. Sample Extraction Procedure

An aliquot of 125 μL of each rat plasma sample (hydrolyzed or unhydrolyzed) was loaded into a Strata™ XA 33 mm polymeric strong anion (30 mg/1 mL) tubes (Phenomenex) and fixed on a complete LiChrolut extraction unit (Merck, Darmstadt, Germany). The cartridge was prior conditioned with 3 mL of acetonitrile and equilibrated with 3 mL of water. Afterward, samples were loaded and washed with 3 mL of methanol: water (30/70, *v*/*v*). Before elution, a 10 min drying step under full vacuum conditions was applied to improve the extraction efficiency. Curcumin was then eluted using 1 mL of formic acid: acetonitrile solution (5:95, *v*/*v*). After elution, the solution was evaporated to dryness using a Techne Sample Concentrator FSC (Scientific Laboratory Supplies, England and Wales) under a stream of nitrogen gas at 45 °C. The dried samples were reconstituted in a 150 μL mobile phase, vortexed for 30 s, and 10 μL was further injected into the chromatograph.

#### 4.7.3. Sample Hydrolyzation

Samples were hydrolyzed to quantify major curcumin metabolites formed after curcumin ingestion (curcumin glucuronide and curcumin sulfate). β-glucuronidase and sulfatase, both from Helix pomatia, were used for curcumin metabolites conversion to their parent curcuminoid. Enzymes were dissolved in 0.1 M phosphate buffer (pH 6.8) to obtain 1800 units of β-glucuronidase and 160 units of sulfatase in 100 μL as described in Mahale et al. [52]. The hydrolysis was performed at 37 °C for 3.5 h by mixing 100 μL β-glucuronidase/sulfatase solution with 100 μL of rat plasma. Before incubation, samples were vortexed for 2 min. The curcumin extraction procedure was performed as previously described.

#### 4.7.4. Identification and Quantification of Curcumin by HPLC-UV

Curcumin from hydrolyzed and unhydrolyzed plasma samples was quantified using the method described by Mahale et al. [52]. Accordingly, an HPLC-UV Waters Alliance 2695 system equipped with a quaternary gradient proportioning valve, autosampler, and degasser, was used. The HPLC system was coupled with a 2489 Waters UV/Visible detector. Separations were performed using a reverse phase-HPLC column (Atlantis^®^ dC18; 4.6 mm × 150 mm, 3 μm particle size, Waters, Hertfordshire, UK) at 25 °C. The chromatographic separation was performed using a mixture of two mobile phases. Mobile phase (A) was 10 mM ammonium acetate (pH 4.5), while mobile phase (B) was acetonitrile. The elution program started from 90% A. The percent of A was further decreased as follows: 90–60% (0–15 min), 60–15% (15–25 min), and 15–0% (25–30 min). After 30 min, the initial conditions were achieved by increasing the percentage of mobile phase A by 90% in 1 min, followed by a 3 min equilibration. The absorbance was recorded at 426 nm. The flow rate was set at 1 mL/min, the injection volume at 10 μL, and the autosampler temperature at 4 °C. Data acquisition and analysis were carried out using the Empower software. The identification of curcumin was carried out using the UV–visible spectra, retention time, co-chromatography with standard, and literature data. The quantity was expressed as μg/mL curcumin equivalents.

### 4.8. Histopathological Examination

Fragments from right-hind paws were taken and fixed in 10% formalin after rats were sacrificed by an overdose of anesthetics. After being embedded in paraffin, fragments were sectioned, stained with hematoxylin and eosin, and examined under a light microscope by a pathologist blinded to the study groups.

### 4.9. Statistical Analysis

Statistica program (v. 13.5, TIBCO Software Inc, Palo Alto, CA, USA) was used for statistical analysis. Multiple comparisons Kruskal–Wallis test was used to assess the differences between groups, followed by posthoc analysis whenever *p* < 0.05. Student *t*-test for independent sample was used to compare the free curcumin and curcumin metabolites between AIcC200 and AInC200 as well as the additional therapeutical effect of cC and nC to D. In the box and whisker plots, the upper line connected with a perpendicular line with the box is the maximum value, the bottom line that is connected with the box represents the minimum value, the upper box edge corresponds to the third quartile, the lower box edge corresponds to the first quartile, line through the center is the median, and × indicates the mean.

## 5. Conclusions

In monotherapy at 200 mg/kg b.w., both conventional curcumin and curcumin nanoparticles have limited analgesic and anti-inflammatory effects in carrageenan-induced paw edema. Curcumin and curcumin nanoparticles associated with diclofenac potentiate its therapeutical effects, with the best results obtained for curcumin nanoparticles. The association of curcumin nanoparticles to a low dose of diclofenac could be an appropriate combination to decrease NSAID doses used to reduce pain, inflammatory cytokines, and histological changes in acute inflammation. Still, additional evaluations are needed to achieve clinical evaluation.

## Data Availability

The presented data will not be publicly available until the associated Ph.D. thesis is publicly defended. Raw data can be obtained upon reasonable request when addressed to Ioana Boarescu (e-mail: ioana.chirila.boarescu@elearn.umfcluj.ro).

## Supplementary Materials

The following supporting information can be downloaded at: https://www.mdpi.com/article/10.3390/ijms231911737/s1.
