# Supplementary material for: Anti-Inflammatory and Analgesic Effects of Curcumin Nanoparticles Associated with Diclofenac Sodium in Experimental Acute Inflammation
Source: Int J Mol Sci. 2022 Oct 3;23(19):11737. doi: 10.3390/ijms231911737 (PMC9570253; doi:10.3390/ijms231911737)
Supplement: Supplementary file 1 [file ijms-23-11737-s001.zip › ijms-1911341-supplementary.pdf]

Supplementary Tables

Anti-Inflammatory and Analgesic Effects of Curcumin Nanoparticles Associated with Diclofenac Sodium in Experimental Acute Inflammation

Ioana Boarescu, Raluca Maria Pop, Paul-Mihai Boarescu, Ioana Corina Bocșan, Dan Gheban, Ruxandra-Mioara Râjnoveanu, Armand Râjnoveanu, Adriana Elena Bulboacă, Anca Dana Buzoianu and Sorana D. Bolboacă

Table S1. Motility test response by groups

Group Abbreviation	1 hour			3 hours			5 hours			7 hours			24 hours		
	Score (n)			Score (n)			Score (n)			Score (n)			Score (n)		
	2	1	0	2	1	0	2	1	0	2	1	0	2	1	0
C	8	0	0	8	0	0	8	0	0	8	0	0	8	0	0
AI	0	4	4	0	4	4	0	1	7	0	2	6	0	4	4
AID	5	3	0	0	5	3	0	4	4	1	7	0	3	5	0
AIcC200	1	5	2	0	5	3	0	3	5	1	3	4	0	6	2
AIcC200D	4	4	0	3	4	1	0	7	1	2	4	2	4	4	0
AInC200	3	5	0	0	7	1	0	4	4	0	6	2	1	5	2
AInC200D	6	2	0	5	2	1	0	8	0	1	7	0	7	1	0

Notes: Data are expressed as absolute frequencies. A score of 2 indicates no motility problems; a score of 1 indicates some motility problems (walk with difficulty, but with the toe of the inflamed paw touching the floor), and a score of 0 indicates motility problems (walk with difficulties and avoided touching the right hind paw to the floor). Abbreviations: C—control; AI—Acute inflammation; D—Diclofenac sodium; cC—conventional curcumin solution (200 mg/kg b.w.); nC—curcumin nanoparticles solution (200 mg/kg b.w.).; n, number – absolute frequency.

Table S2. Nociceptive thresholds by groups on paw pressure test.

Group Abbreviation	1 hour	3 hours	5 hours	7 hours	24 hours
C	7.4 (0.9)	7.2 (0.7)	7.4 (0.6)	7.5 (0.7)	7.5 (0.8)
AI	6.1 (0.8)	3.9 (0.4)	2.7 (0.3)	3.7 (0.4)	5.1 (0.2)
AID	6.6 (0.6)	4.8 (0.5)	3.7 (0.4)	4.7 (0.5)	6.2 (0.5)
AIcC200	6.3 (0.8)	4 (0.3)	3.3 (0.3)	4.1 (0.5)	5.5 (0.8)
AIcC200D	6.7 (0.3)	5.2 (0.2)	4.3 (0.5)	5.4 (0.4)	6.3 (0.4)
AInC200	6.2 (0.4)	4.3 (0.4)	3.6 (0.5)	4.4 (0.4)	5.8 (0.4)
AInC200D	6.8 (0.4)	5.6 (0.5)	5.1 (0.4)	5.8 (0.5)	6.7 (0.4)

Notes: values expressed as mean (standard deviation), n=8 for each group. Abbreviations: C, control; AI, Acute inflammation; D, Diclofenac ; cC, conventional curcumin solution (200 mg/kg b.w.); nC, curcumin nanoparticles solution (200 mg/kg b.w.).

Table S3. Comparisons between nociceptive thresholds by groups expressed as p-values.

Group Abbreviation	1 hour	3 hours	5 hours	7 hours	24 hours
AI vs.					
C	0.0649	<0.0001	<0.0001	<0.0001	<0.0001
AID vs.					
C	>0.9999	0.0753	0.0165	0.0224	0.5845
AI	>0.9999	0.5731	0.2979	0.4246	0.0753
AIcC200 vs.					
C	0.0633	<0.0001	0.0002	0.0001	0.0009
AI	>0.9999	>0.9999	>0.9999	>0.9999	>0.9999
AID	>0.9999	0.8711	>0.9999	>0.9999	>0.9999
AIcC200D vs.					
C	>0.9999	>0.9999	0.6693	>0.9999	>0.9999
AI	>0.9999	0.0285	0.0052	0.0037	0.0270
AID	>0.9999	>0.9999	>0.9999	>0.9999	>0.9999
AIcC200	>0.9999	0.0505	0.4160	0.1400	0.5845
AInC200 vs.					
C	0.0682	0.0007	0.0046	0.0016	0.0190
AI	>0.9999	>0.9999	0.7224	>0.9999	>0.9999
AID	>0.9999	>0.9999	>0.9999	>0.9999	>0.9999
AInC200D vs.					
C	>0.9999	>0.9999	>0.9999	>0.9999	>0.9999
AI	0.9205	0.0033	<0.0002	0.0005	0.00120
AID	>0.9999	>0.9999	0.5296	>0.9999	>0.9999
AInC200	0.9547	0.1056	0.2104	0.1968	0.5403

Notes: Abbreviations: AI, Acute inflammation; D, Diclofenac; cC, conventional curcumin solution (200 mg/kg b.w.); nC, curcumin nanoparticles solution (200 mg/kg b.w.).

Table S4. Reactions time to the heat in hot plate test, by groups.

Group Abbreviation	1 hour	3 hours	5 hours	7 hours	24 hours
C	9.8 (1.1)	9.9 (1.1)	10.2 (1.1)	9.6 (1)	9.9 (0.9)
AI	6.5 (0.5)	5.1 (0.5)	3.2 (0.4)	3.9 (0.3)	5.8 (0.9)
AID	7.1 (0.6)	7.3 (0.7)	6.8 (0.8)	6.6 (0.4)	7 (0.6)
AIcC200	6.3 (0.6)	5.4 (0.5)	4.2 (0.3)	5.2 (0.4)	6.3 (0.6)
AIcC200D	7.3 (0.5)	7.8 (0.3)	7.7 (0.4)	7.3 (0.6)	7.3 (0.5)
AInC200	6.5 (0.6)	6.5 (0.5)	5.7 (0.4)	6.2 (0.5)	6.6 (0.4)
AInC200D	7.7 (0.7)	8.8 (0.7)	8.6 (0.5)	8.7 (0.5)	8.4 (0.6)

Notes: values expressed as mean (standard deviation), n=8 for each group. Abbreviations: C, control; AI, Acute inflammation; D, Diclofenac ; cC, conventional curcumin solution (200 mg/kg b.w.); nC, curcumin nanoparticles solution (200 mg/kg b.w.).

Table S5. Comparisons expressed as p-values between reactions time to the heat, in hot plate, by groups.

Abbreviations	1 hour	3 hours	5 hours	7 hours	24 hours
AI vs.					
C	0.0002	<0.0001	<0.0001	<0.0001	<0.0001
AID vs.					
C	0.1246	0.1925	0.1082	0.1275	0.0260
AI	>0.9999	0.1334	0.0545	0.0772	>0.9999
AIcC200 vs.					
C	<0.0001	<0.0001	<0.0001	<0.0001	<0.0001
AI	>0.9999	>0.9999	>0.9999	>0.9999	>0.9999
AID	>0.9999	0.3832	0.7938	>0.9999	>0.9999
AIcC200D vs.					
C	0.3175	0.9722	>0.9999	>0.9999	0.2463
AI	0.9547	0.0175	0.0025	0.0036	0.2256
AID	>0.9999	>0.9999	>0.9999	>0.9999	>0.9999
AIcC200	0.4891	0.0618	0.0753	0.1336	0.8122
AInC200 vs.					
C	0.0006	0.0052	0.0031	0.0102	0.0014
AI	>0.9999	>0.9999	0.9205	0.6441	>0.9999
AID	>0.9999	>0.9999	>0.9999	>0.9999	>0.9999
AInC200D vs.					
C	>0.9999	>0.9999	>0.9999	>0.9999	>0.9999
AI	0.0735	0.0001	<0.0001	<0.0001	0.0013
AID	>0.9999	>0.9999	>0.9999	0.5845	0.5496
AInC200	0.1535	0.0893	0.1056	0.0682	0.0604

Notes: Abbreviations: AI, Acute inflammation; D, Diclofenac ; cC, conventional curcumin solution (200 mg/kg b.w.); nC, curcumin nanoparticles solution (200 mg/kg b.w.).

Table S6. Pro-inflammatory cytokines for plasma and tissue levels by groups.

Group Abbreviation	TNF-α [pg/mL]		IL-6 [pg/mL]		IL-1β [pg/mL]	
	plasma	tissue	plasma	tissue	plasma	tissue
C	60.9 (14.1)	125.4 (31.9)	130.2 (13.1)	123.1 (18)	173.9 (9.9)	197.1 (15)
AI	126.5 (14.8)	546.5 (107.5)	323.3 (58.7)	486.6 (61.7)	381.5 (49)	548 (64.3)
AID	79.5 (9.8)	257 (87.9)	212.1 (16.3)	289 (16.9)	243.9 (11.8)	331.5 (18.4)
AIcC200	120.2 (9.6)	284 (51.9)	247.4 (38.4)	353.7 (31)	321 (37.6)	407.8 (69.3)
AIcC200D	88.7 (13.2)	226 (58.3)	161.9 (12.4)	228.1 (16.8)	220.8 (22.7)	281.5 (30.3)
AInC200	93.7 (11.5)	257 (29.6)	171.7 (18.4)	295.2 (39.8)	270.2 (27.6)	330.3 (49)
AInC200D	68.1 (8.6)	132.4 (21.1)	138.5 (14.9)	191.9 (31.7)	203.3 (11.2)	214 (23.3)

Notes: values expressed as mean (standard deviation), n=8 for each group. Abbreviations: TNF-α, Tumoral necrosis α; IL-6, Interleukin 6 ; IL-1β, Interleukin 1β; C, control; AI, Acute inflammation; D, Diclofenac ; cC, conventional curcumin solution (200 mg/kg b.w.); nC, curcumin nanoparticles solution (200 mg/kg b.w.).

Table S7. Comparisons expressed as p-values between pro-inflammatory cytokines for plasma and tissue levels, by groups.

Group Abbreviation	TNF-α [pg/mL]		IL-6 [pg/mL]		IL-1β [pg/mL]	
	plasma	tissue	plasma	tissue	plasma	tissue
AI vs.						
C	<0.0001	<0.0001	<0.0001	<0.0001	<0.0001	<0.0001
AID vs.						
C	>0.9999	0.1082	0.0052	0.0161	0.0531	0.0293
AI	0.0170	0.1643	>0.9999	0.2454	0.1400	0.3753
AIcC200 vs.						
C	<0.0001	0.0044	0.0002	<0.0001	<0.0001	0.0003
AI	>0.9999	>0.9999	>0.9999	>0.9999	>0.9999	>0.9999
AID	0.0380	>0.9999	>0.9999	>0.9999	>0.9999	>0.9999
AIcC200D vs.						
C	0.3242	0.6318	>0.9999	>0.9999	0.9722	>0.9999
AI	0.2508	0.0212	0.0077	0.0016	0.0039	0.0054
AID	>0.9999	>0.9999	>0.9999	>0.9999	>0.9999	>0.9999
AIcC200	0.4699	>0.9999	0.1466	0.0872	0.0717	0.2916
AInC200 vs.						
C	0.1275	0.0175	0.8240	0.0097	0.0041	0.0195
AI	0.5960	0.7224	0.02300	0.3600	0.9374	0.5090
AID	>0.9999	>0.9999	>0.9999	>0.9999	>0.9999	>0.9999
AInC200D vs.						
C	>0.9999	>0.9999	>0.9999	>0.9999	>0.9999	>0.9999
AI	<0.0002	<0.0001	<0.0001	<0.0001	0.0001	<0.0001
AID	>0.9999	0.1570	0.0212	0.5193	>0.9999	0.1644
AInC200	0.3453	0.0270	>0.9999	0.3600	0.2508	0.1161

Notes: Abbreviations: TNF-α, Tumoral necrosis α; IL-6, Interleukin 6; IL-1β, Interleukin 1β; C, control; AI, Acute inflammation; D, Diclofenac; cC, conventional curcumin solution (200 mg/kg b.w.); nC, curcumin nanoparticles solution (200 mg/kg b.w.).
